# Supplementary material for: Structural basis of subtype-selective competitive antagonism for GluN2C/2D-containing NMDA receptors
Source: Nat Commun. 2020 Jan 22;11:423. doi: 10.1038/s41467-020-14321-0 (PMC6976569; doi:10.1038/s41467-020-14321-0)
Supplement: Supplementary file 1 — Supplementary Information [file 41467_2020_14321_MOESM1_ESM.pdf]

Supplementary Information

Structural basis of subtype-selective competitive antagonism for GluN2C/2D-containing NMDA receptors

Authors: Jue Xiang Wang, Mark W. Irvine, Erica S. Burnell, Kiran Sapkota, Robert J. Thatcher, Minjun Li, Noriko Simorowski, Arturas Volianskis, Graham L. Collingridge, Daniel T. Monaghan, David E. Jane, Hiro Furukawa

This file contains:

Supplementary Note 1

Supplementary Table 1-2

Supplementary Figures 1-8

Supplementary Methods

Supplementary References

Supplementary Note 1

Study of the HQA-binding site in the GluN2 LBD

HQA is a derivative of the endogenous NMDAR agonist quinolinate and has lower potencies than glutamate across all GluN2 subunits. EC_{50} values determined by TEVC range from 10-20 μM for GluN2A- and GluN2B-containing receptors, and 30-90 μM for GluN2C- and GluN2D-containing receptors[1-3]. We obtained the GluN1/GluN2A and GluN1/GluN2A-4m LBD heterodimers in complex with glycine and HQA at 1.9 Å (Supplementary Table 1). The structures were solved by molecular replacement using the glycine/glutamate-bound GluN1-GluN2A LBD heterodimer (PDB: 4NF8)[4]. The structures showed the HQA-binding mode in NMDARs for the first time and allowed comparison of the Gly/Glu *vs* Gly/HQA-bound structures for both GluN1/GluN2A-4m (Supplementary Fig. 7) and GluN1/GluN2A LBD (Supplementary Fig. 8).

In both structures, the α-carboxyl group of glutamate and the 2-carboxyl group of HQA form hydrogen bonds with residues Arg518, Ser689, and Thr513. The γ-carboxyl group of glutamate and the 3-carboxymethyl group of HQA show some subtle differences: both form hydrogen bonds with Thr690, but the γ-carboxyl group of glutamate additionally interacts with Asp731 and is positioned to form hydrogen bonds to W2 and W3. In contrast, HQA only forms hydrogen bonds to the equivalent W2', whereas W3' is not part of this interaction network (but forms hydrogen bonds with other residues, excluded for clarity). The amine group of glutamate can form interactions to another water (W1) as well as hydrogen bonds to Thr513 and Ser511, none of these interactions are present in the HQA-bound structure. There might be, however, some favorable π-π-stacking interactions between the pyrimidine ring of HQA and His484 (3.5 Å distance).

As described above, glutamate is able to form more hydrogen bonds with the residues of the binding pocket compared to HQA. There are also two further key differences between glutamate- and HQA-bound GluN2A (or GluN2A-4m) LBD binding pockets, which likely change interactions between the D1 and D2 lobes, thereby affecting the stability of the closed LBD clamshell conformation: 1) the availability of the water W1 seen only in glutamate-bound structures and that bridges D1 (Glu413) and D2 residues (Tyr761, Asp731) (Supplementary Fig. 7a and 8a) (note that W1 is in a position to form up to four hydrogen bonds); and 2) there is a positional shift of Tyr730. In the glutamate-bound structure, Tyr730 forms a direct hydrogen bond with Glu413, resulting in strengthening of the D1-D2 interaction. In contrast, the Tyr730 side chain in the HQA-bound structures is oriented in a different direction to accommodate the bulky pyridine ring of HQA, (Supplementary Fig. 7c and 8c). Hence, in the HQA-bound structure, the Tyr730-Glu413 interaction is lost. Overall, the above structures show that the weaker potency and efficacy of HQA are mediated by the less stable D1-D2 interactions of the LBD bilobes compared to the glutamate-bound form.

Supplementary Table 1. Data collection and refinement statistics for HQA-bound structures

	GluN1/GluN2A LBD +Gly, HQA	GluN1/ GluN2A-4m LBD + Gly, HQA
**Data collection**		
Space group	$P2_12_12_1$	$P2_12_12_1$
Cell dimensions		
a, *b*, *c* (Å)	54.37, 88.72 125.48	55.02, 89.86, 124.58
α, β, γ (°)	90, 90, 90	90, 90, 90
Resolution (Å)	46.36-1.87 (1.91-1.87)	72.88-1.94 (1.97-1.94)
R_{merge}	0.116 (0.944)	0.123 (0.885)
I /σ	9.5 (2.1)	9.2 (2.0)
Completeness (%)	99.9 (99.9)	99.4 (99.9)
Redundancy	6.8 (7.1)	6.2 (6.2)
**Refinement**		
Resolution (Å)	46.36-1.87	50.33 – 1.94
No. reflections	50,584 (4950)	46,159 (4559)
R_{work} / R_{free}	0.1727 / 0.2075	0.2107 / 0.2501
No. non-H atoms		
Protein	4546	4518
Ligands	18	18
Water	405	288
B-factors		
Protein	31.51	44.51
Ligands	19.80	27.28
Water	38.35	47.10
R.m.s. deviations		
Bond lengths (Å)	0.007	0.010
Bond angles (°)	0.834	0.960
Ramachandran		
Favored (%)	96.61	96.24
Allowed (%)	3.04	3.58
Outliers (%)	0.36	0.18

Each dataset was collected from a single crystal at 0.92012 Å. Values in parentheses are for highest-resolution shell. GluN2A-4m refers to GluN2A with 4 residue mutations: Ala414Arg, Lys738Met, Gly740Arg, Arg741Lys.

Supplementary Table 2. Effect of antagonist compounds on GluA2 and GluK2. Addition of 30 and 100 µM of the tested antagonist compounds (UBP791, UBP1700-02) showed minimal effects on glutamate-stimulated Ca^{2+}-influx in HEK293 cells expressing either human GluA2 or GluK2. As all tested compounds and concentrations led to at least 85% of the normalized response, IC_{50} values were at least 100 µM. Mean response and standard error of the mean are as listed.

UBP791:

GluA1, IC_{50} > 100 µM, n = 4

Concentration (µM)	Mean Response	SEM
100	86.0	4.5
30	94.1	3.1

GluK2, IC_{50} > 100 µM, n=3

Concentration (µM)	Mean Response	SEM
100	97.8	1.2
30	96.1	0.3

UBP1700

GluA1, IC_{50} > 100 µM , n=3

Concentration (µM)	Mean Response	SEM
100	101.8	2.4
30	105.1	1.4

GluK2, IC_{50} > 100 µM, n=3

Concentration (µM)	Mean Response	SEM
100	90.4	7.9
30	76.5	12.3

UBP1701

GluA1, IC_{50} > 100 µM, n=3

Concentration (µM)	Mean Response	SEM
100	91.6	3.6
30	92.5	2.0

GluK2, IC_{50} > 100 µM, n=3

Concentration (µM)	Mean Response	SEM
100	96.1	1.0
30	96.9	3.0

UBP1702

GluA1, IC_{50} > 100 µM, n=3

Concentration	Mean Response	SEM
100	97.4	4.1
30	86.5	1.5

GluK2, IC_{50} > 100 µM, n=3

Concentration	Mean Response	SEM
100	96.0	1.6
30	94.5	4.4

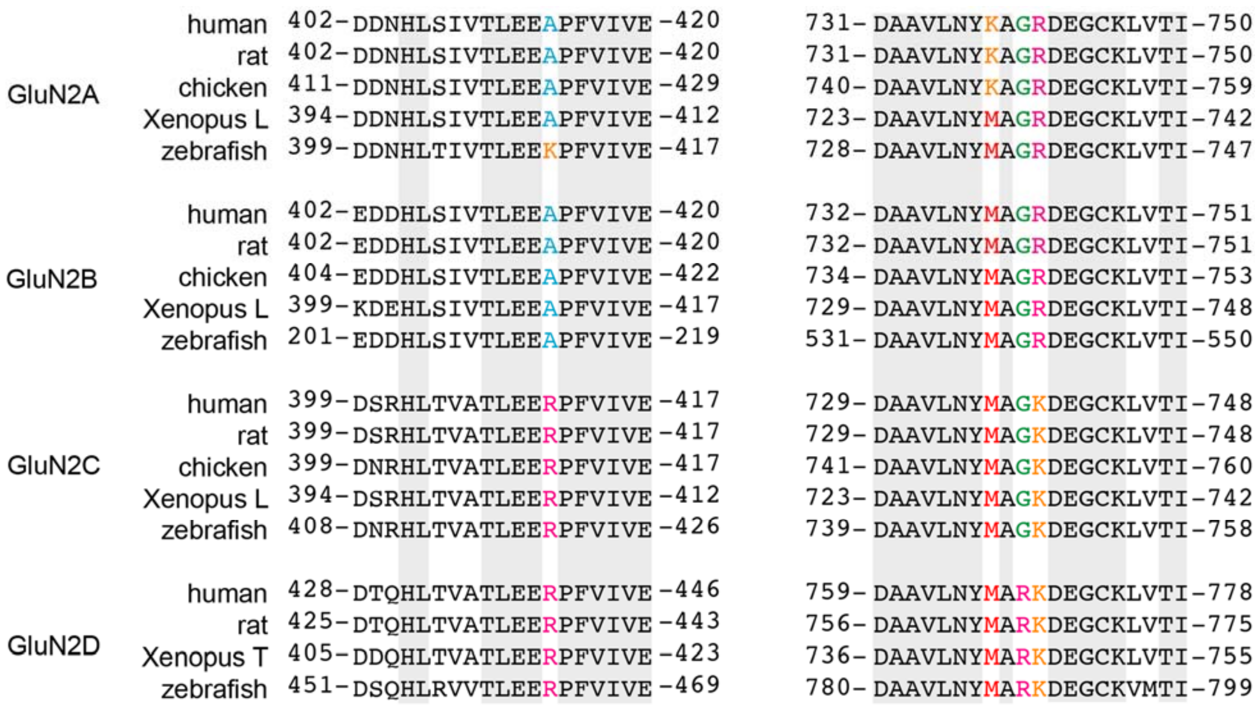

Supplementary Figure 1. Primary sequence alignment of GluN2 subunits across different species. The sites of the four mutations (colored) in Fig. 3b are highly conserved across various species (shown here: Rattus norvegicus (rat), Gallus gallus (chicken), Xenopus laevis or tropicalis (frog), Danio rerio (zebrafish)). An exception is seen in GluN2A where two of the four sites are not conserved in *Xenopus laevis* and zebrafish, respectively.

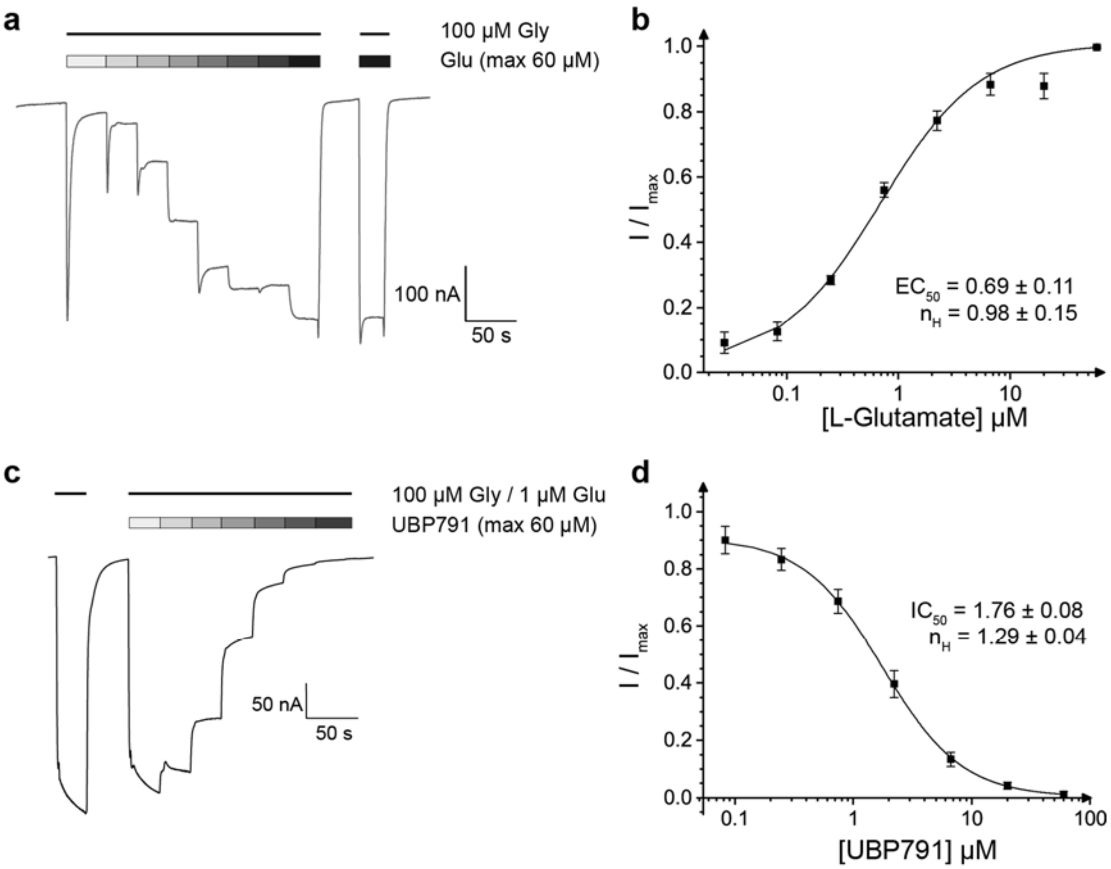

Supplementary Figure 2. Dose-response traces and EC_{50}/IC_{50} curves of GluN1-4a/GluN2A-4m. a Representative TEVC dose-response recordings of GluN1-4a/GluN2A-4m NMDARs held at -60 mV. Currents were evoked by application of 100 µM glycine and varying concentrations of L-glutamate (three-fold increment from 0.027-60 µM). **b** Averaged dose-response curves (mean ± standard deviation) for activation with L-glutamate from six GluN1-4a/GluN2A-4m recordings fit with the Hill equation to calculate EC_{50} value for averaged curve and Hill coefficient (n_H). **c** Representative TEVC dose-response traces of GluN1-4a/GluN2A-4m NMDARs held at -60 mV. Currents were evoked by application of 100 µM glycine and 1 µM glutamate and inhibited by varying concentrations of UBP791 (max 60 µM, concentration increments: 0.12 / 0.37 / 1.1 / 3.3 / 10 / 30 / 60 µM). **d** Averaged dose-response curves (mean ± standard deviation) for inhibition by UBP791 from seven GluN1-4a/GluN2A-4m recordings fit with the Hill equation to calculate IC_{50} value from averaged curve and Hill coefficient (n_H).

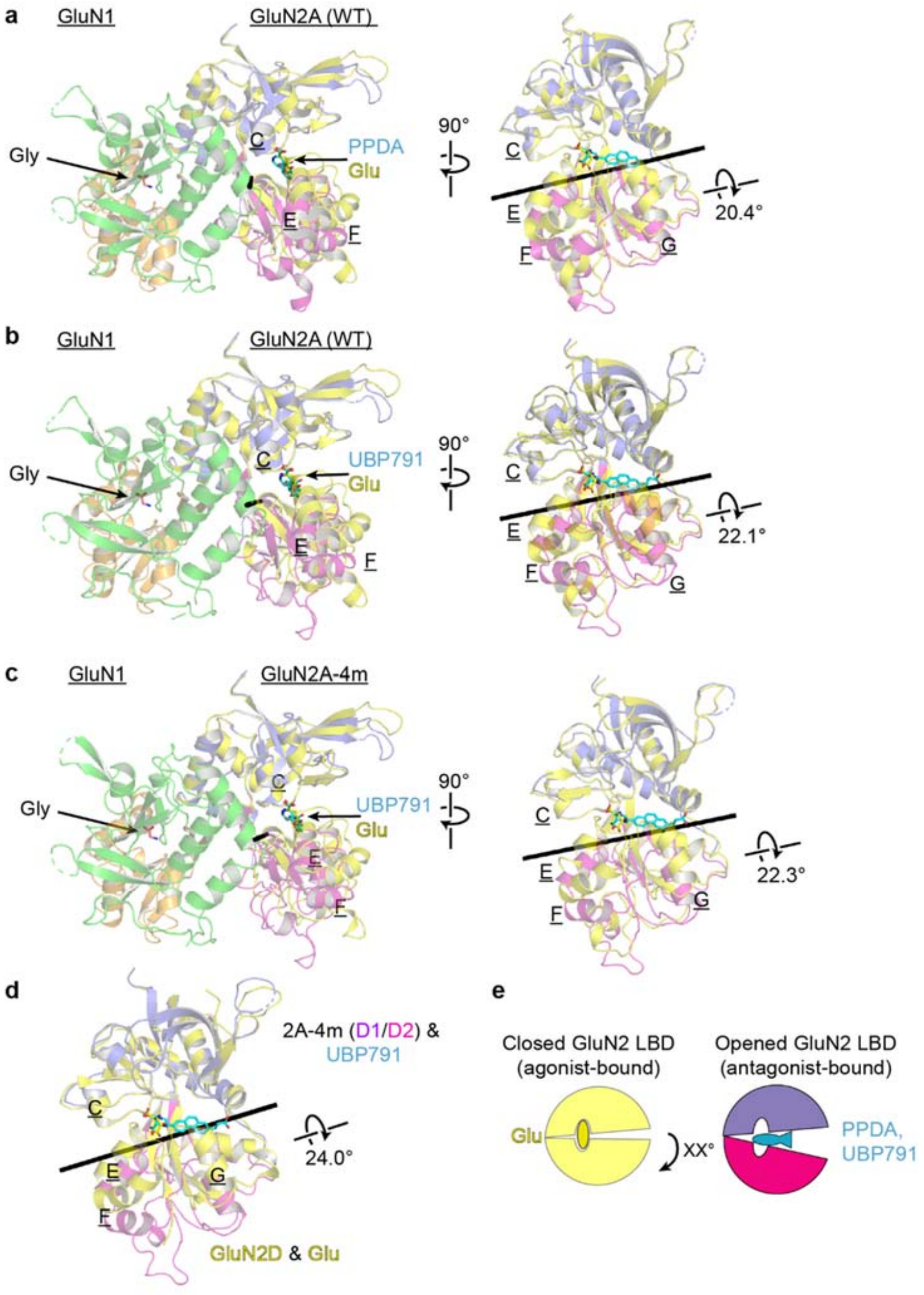

Supplementary Figure 2. Domain opening of LBDs from agonist-bound state to antagonist-bound state. While the bilobe architecture of the agonist (glutamate)-bound GluN2 LBD is 'closed,' binding of the antagonist (PPDA or UBP791) 'opens up' the LBD by rotating the GluN2 D2 lobe. The GluN2 agonist-bound state is depicted in yellow, while the antagonist-bound GluN2 D1 and D2 are colored purple or magenta, respectively. The figures show the degree of opening upon binding of PPDA in GluN2A (PDB: 4NF8, 4NF6) (**a**), binding of UBP791 in GluN2A (PDB: 4NF8 *vs* Gly/UBP791-bound GluN1-GluN2A LBD structure) (**b**), binding of UBP791 in GluN2A-4m (*vs* the Gly/Glu-bound GluN1-GluN2A-4m LBD) (**c**), and binding of UBP791 in GluN2A-4m compared to the Glu-bound GluN2D structure (PDB: 3OEN) (**d**). **e** Schematic representation of the antagonist-induced domain opening by rotation of D2.

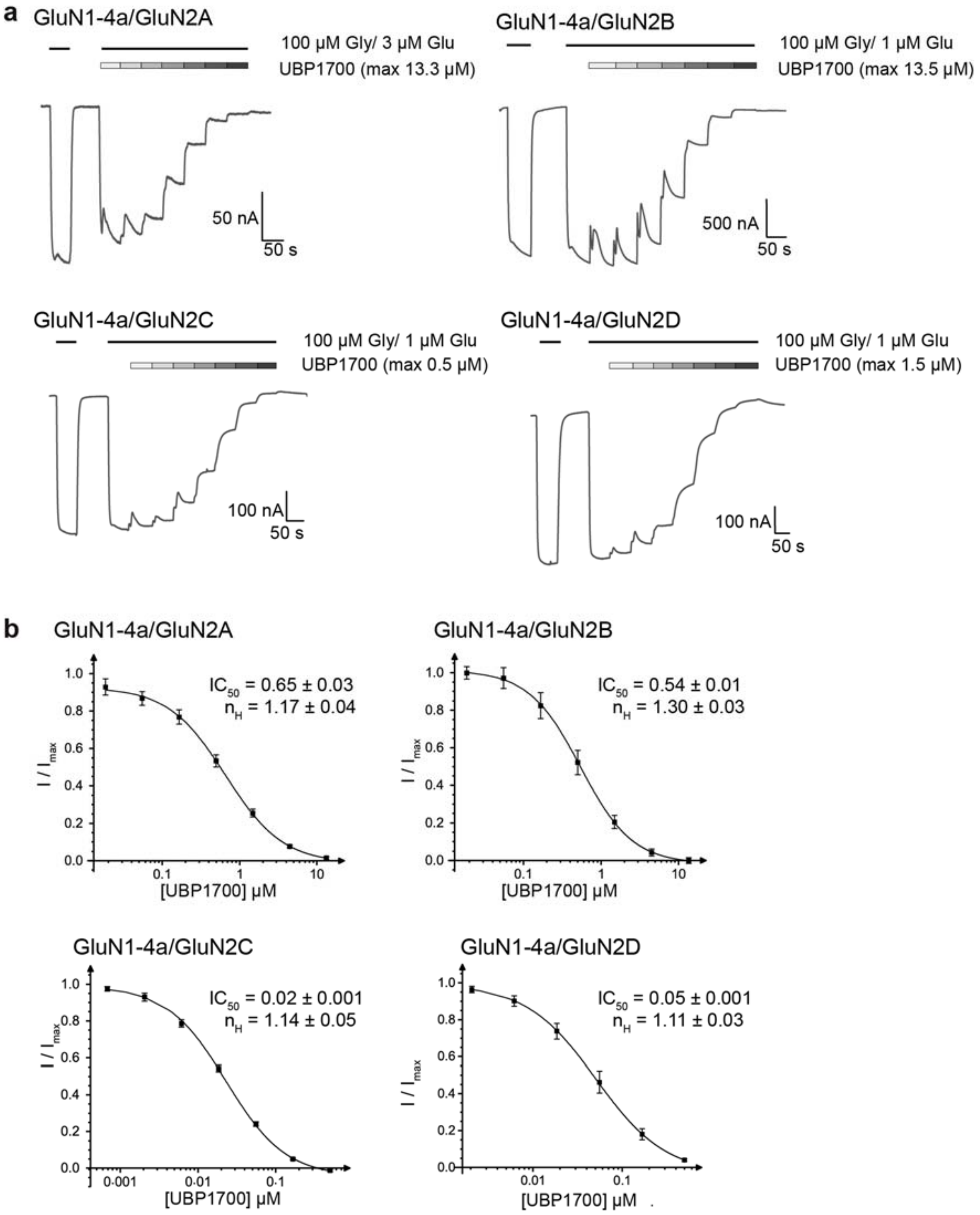

Supplementary Figure 4. Subtype-selectivity of UBP1700. a Representative TEVC dose-response recordings of NMDARs composed of GluN1-4a and GluN2A/B/C/D held at -60 mV. Currents were evoked by application of 100 μM glycine and 1 or 3 μM glutamate, and inhibited by varying concentrations of UBP1700 (three-fold increments with max UBP1700 concentration as shown). **b** Averaged dose-response curves (mean ± standard deviation) for inhibition with UBP1700 from eight, six, eight, and six recordings of the GluN2A, GluN2B, GluN2C, and GluN2D subtypes, respectively, fit with the Hill equation to calculate IC_{50} values from averaged curves and Hill coefficients (n_H).

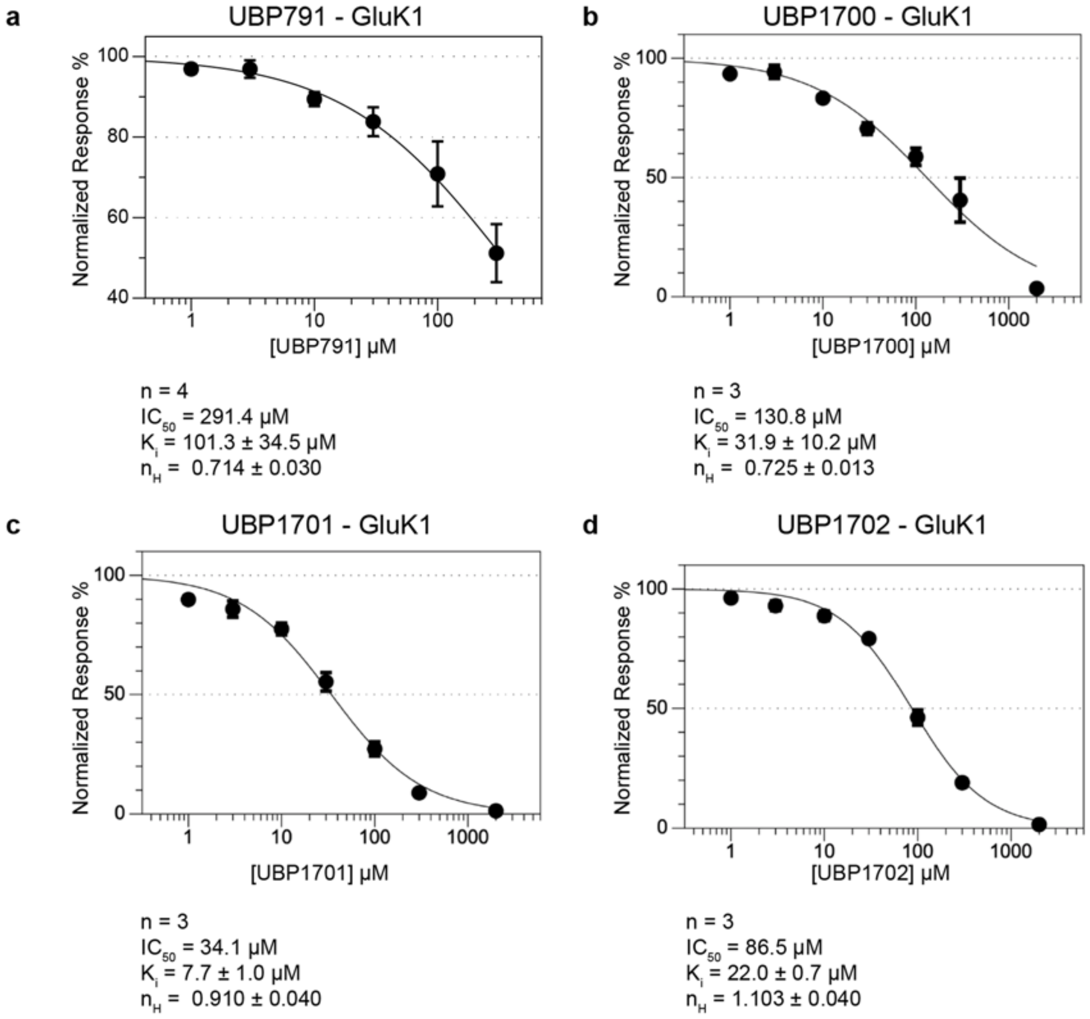

Supplementary Figure 5. Effect of antagonist compounds on GluK1.
Averaged dose-response curves (average ± standard error of the mean) for inhibition with **a** UBP791, **b** UBP1700, **c** 1701, or **d** 1702 on human GluK1 expressed in HEK293 cells in the glutamate-stimulated Ca^{2+}-influx assay from 3 or 4 measurements (n). IC_{50}, K_i, and Hill coefficient n_H (mean ± standard error of the mean) calculated with the Cheng-Prusoff equation were determined per measurement and are as shown.

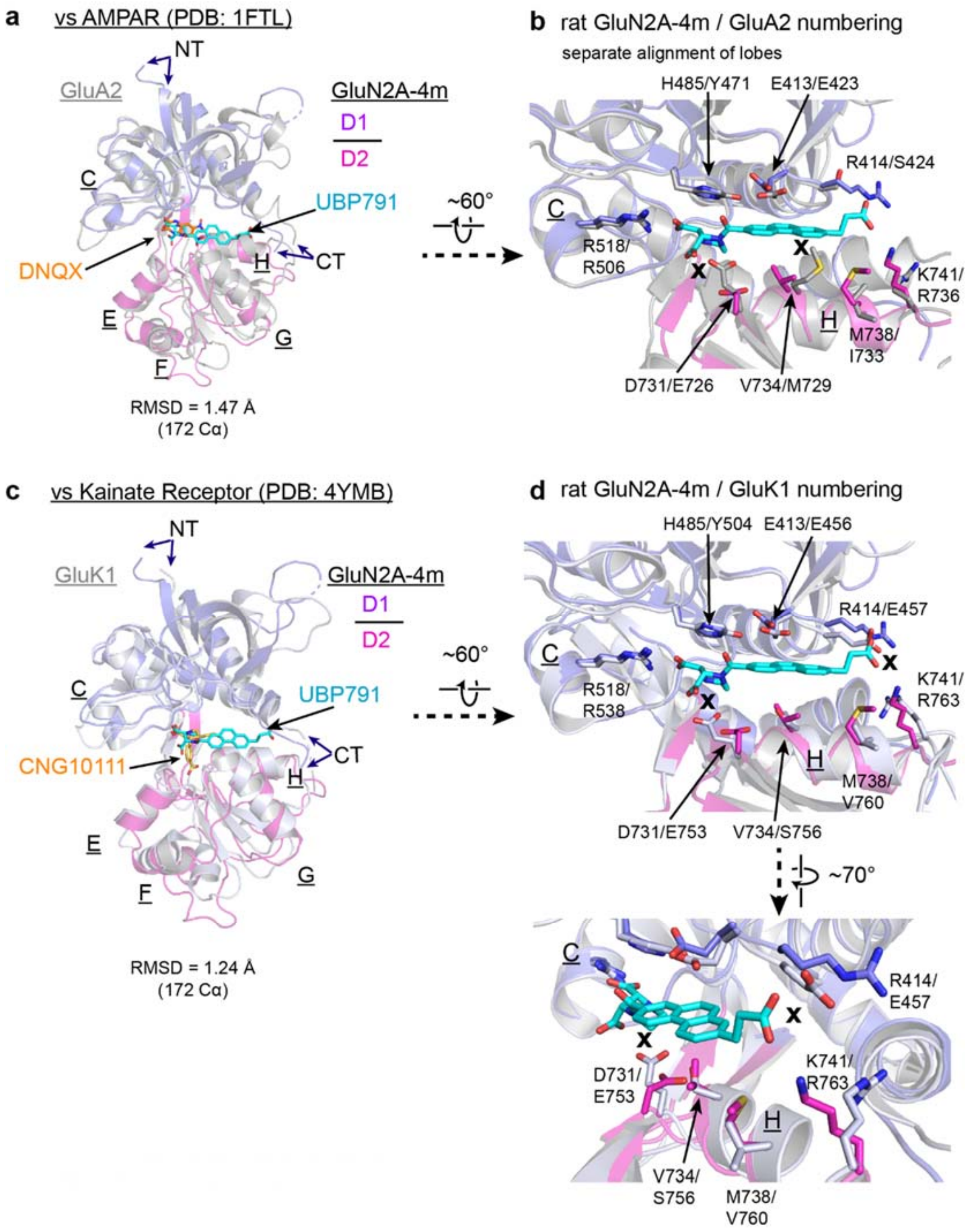

Supplementary Figure 6. Modeling binding site of UBP791 in non-NMDARs. a Superposition of the GluN2A-4m LBD in complex with UBP791 (cyan sticks) with the GluA2 AMPA receptor LBD in complex with antagonist DNQX (yellow sticks) (PDB: 1FTL). **b** Separate alignment of GluA2 LBD D1 and D2 lobes with UBP791-bound GluN2A-4m LBD D1 and D2 show that GluA2-Glu726/Met729 may sterically clash with UBP791 (marked with 'X'). **c** Superposition of the GluN2A-4m LBD in complex with UBP791 (cyan) with the GluK1 Kainate receptor LBD in complex with antagonist CNG10111 (yellow) (PDB: 4YMB). **d** GluK1-Glu753 potentially clashes with UBP791 and GluK1-Glu457 and the carboxyethyl group of UBP791 could have charge repulsion (both marked with 'X'). GluA2 and GluK1 are in grey, helices in the NMDAR LBDs are labelled with underlined letters and color-coded in accordance to Fig. 1.

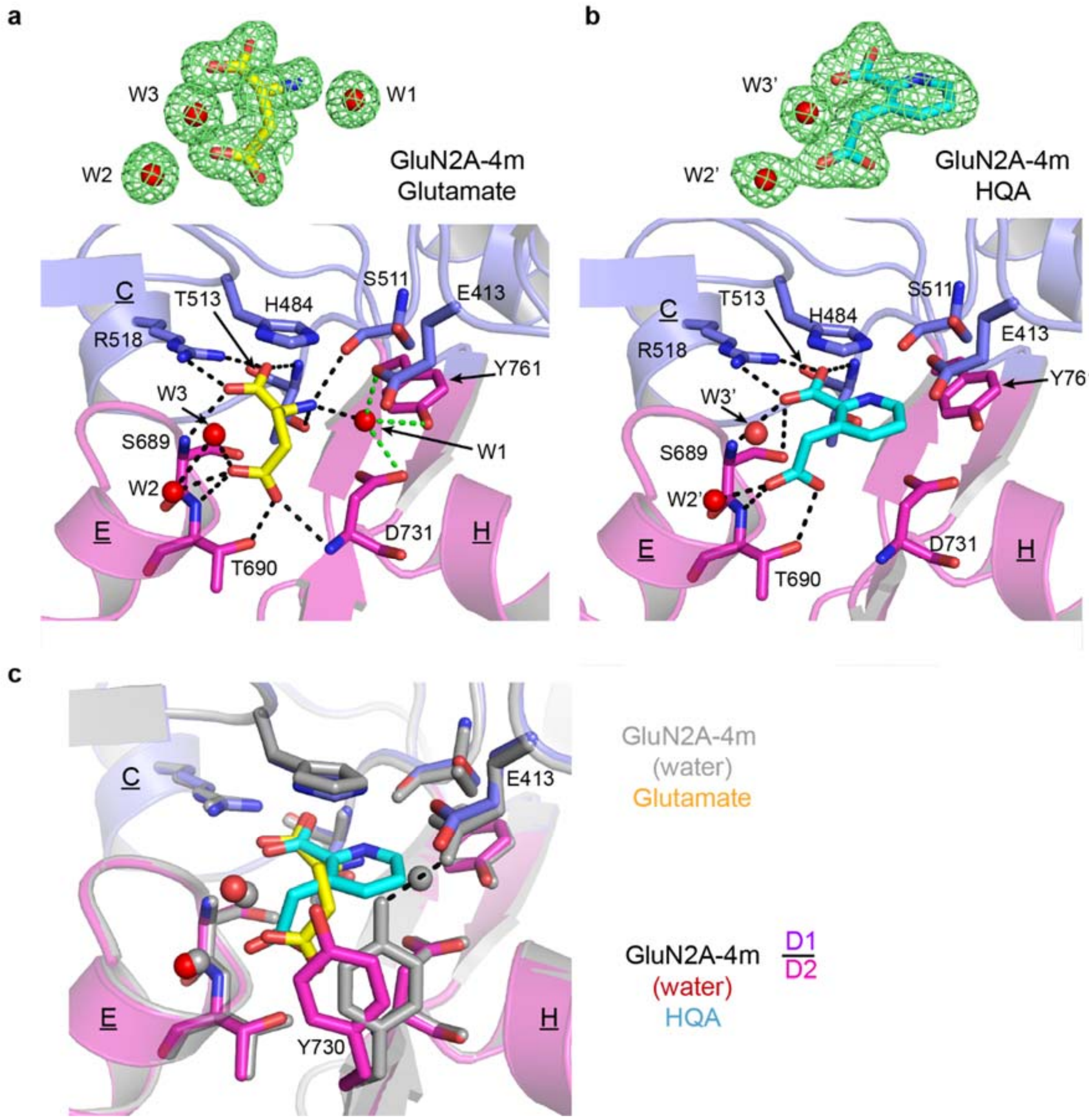

Supplementary Figure 7. Glutamate or HQA-binding site in GluN2A-4m LBD. (a-b) Presented are the binding sites of glutamate (yellow sticks) or HQA (cyan sticks) within GluN2A-4m at the cleft between D1 (purple) and D2 (pink). Shown in mesh is the F_o-F_c omit map of glutamate or HQA contoured at 3σ (top panel). **(a)** Glutamate forms hydrogen bonds (black dashes) with multiple residues from the D1 and D2 lobe (purple and pink sticks), as well as three waters W1-W3 (red spheres). In particular, W1 is able to form hydrogen bonds (green dashes) with D1 and D2 lobe residues. **(b)** HQA forms similar hydrogen bonds with GluN2A-4m residues (black dashes), potentially a π- π interaction with His484, and a hydrogen bond with W2'. W3' is not interacting with other depicted elements, but presented for comparison. **(c)** Overlay of two binding sites, with glutamate-bound GluN2A-4m in grey, and HQA-bound GluN2A-4m is colored as before (purple/pink). Notably, there is no equivalent W1 in the HQA-bound structure, and the position of Tyr730 changes, while all other residues overlap well. Tyr730 and Glu413 in the glutamate-bound structure (grey) can form a direct hydrogen bond (does not involve W1).

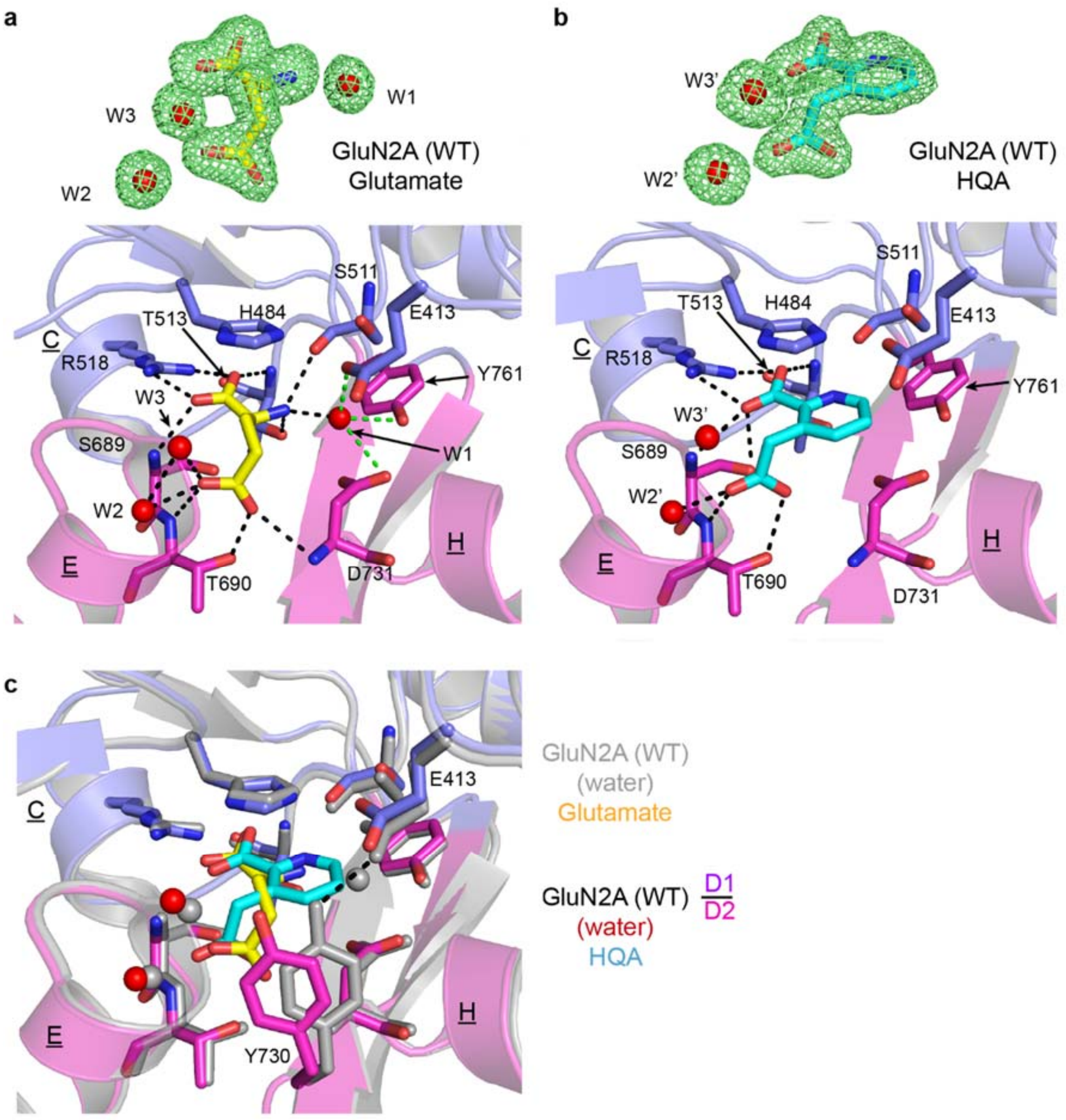

Supplementary Figure 8. Glutamate or HQA-binding site in GluN2A LBD. (a-c) Structures of Gly/Glu-bound and Gly/HQA-bound GluN1/GluN2A LBDs viewed from the similar angle to that in Supplementary Fig. 7. The Gly/Glu-bound structure (PDB: 4NF8) is from Jespersen et al, 2014. Shown in mesh is the F_o-F_c omit map of glutamate or HQA contoured at 3σ (top panel).

Supplementary Methods

Cell Culture

HEK293 cell lines stably expressing homomeric glutamate receptors (GluA1, GluK1 and GluK2) were obtained from Eli Lilly and grown in DMEM (ThermoFisher) supplemented with 10% *v/v* fetal bovine serum (Sigma), 2 mM Glutamax™ (Gibco), 100 U/mL penicillin, 100 µg streptomycin and 250 ng amphotericin B (Sigma) in 5% CO_2 at 37 °C. Cells were passaged using TrypLE (Gibco) as required before confluency and periodically treated with selection antibiotics (GluK1, GluK2 hygromycin B, 200 µg/mL; GluA1 geneticin™, 200 µg/mL).

Calcium influx fluorescence assay

100k cells/well were seeded into a 96-well plate precoated with poly-L-lysine and incubated for 12-16 hours until fully confluent. The cell monolayer was washed twice with 100 µL Hanks Balanced Salt Solution supplemented with 5 mM $CaCl_2$ and 20 mM HEPES (sHBSS), then incubated for 2-4 hours with 100 µL working solution of Ca6 dye (Molecular Devices) supplemented with a desensitization blocker (GluK1, GluK2 0.25 mg/mL concanavalin A, Sigma; GluA1 100 µM cyclothiazide, Sigma). The time resolved fluorescence was recorded in a Flexstation 3 instrument (Molecular Devices). For each individual reading, the initial fluorescence of the well was measured for 20 seconds, after which the desired concentration of test antagonist was added followed by addition of the L-glutamate agonist 70 seconds later. The response is measured as the peak change in fluorescence upon addition of agonist. The viability of the cells was confirmed by determination of the concentration-response curve to the L-glutamate agonist and a standard antagonist for each plate. Readings of antagonists used the experimentally determined EC_{80} value of L-glutamate. Fluorescence traces were performed in

triplicate on each plate and K_i values were determined on a plate-by-plate basis. Separate experiments (n) were performed on different passages of cells.

Synthesis of compounds UBP791 and UBP1700-1702

Here, we describe a detail of chemical synthesis of UBP791 and UBP1700-1702[5,6]. A series of N^1-substituted piperazine-2,3-dicarboxylic acids derivatives (UBP791, 1700, 1701, 1702) were synthesized by reacting the acid chlorides of various carboxylic acids (**11**, **12**, **15**, **17**) with the cis-isomer of piperazine-2,3-dicarboxylic acid under modified Schotten-Baumann conditions (Scheme 1). Synthesis of the carboxylic acids required for coupling began with **1** which was subjected to Friedel-Crafts acylation to afford the 2-acyl derivative **2** (Scheme 2). A haloform reaction was utilised to conveniently convert the acetyl to the corresponding acid **3** which was subsequently brominated at the 7-position to yield **4** (Scheme 2). Unfortunately, attempts to oxidize **4** to the corresponding phenanthrene failed due to poor solubility. To get around this problem, **4** was converted to its methyl ester **5** via Fischer esterification and then oxidized to phenanthrene **6** smoothly using NBS and benzoyl peroxide (Scheme 2). Base hydrolysis of **6** afforded acid **7** which was then re-esterified to either a *t*-butyl (**8**) or benzyl (**9**) ester. The desired acidic side-chains were introduced to the 7-position of **8** or **9** via Heck coupling (Scheme 3 & 4). Reaction of **8** with ethyl acrylate yielded **10** which could be selectively de-protected at the 2-position via reaction with TFA (Scheme 3). Whilst alkene acid **11** was utilised for coupling to form UBP1700, some was held back and hydrogenated to form the corresponding alkyl acid **12** which was used in the synthesis of UBP791 (Schemes 1 & 3). Heck coupling between **8** and ethyl methacrylate yielded **13** which was subsequently hydrogenated to **14** (Scheme 4). Selective de-protection at the 2-position to afford **15** was achieved via reaction with TFA. Alkene **16** was

generated via Heck coupling between ethyl 4-pentenoate and **9** (Scheme 4). Removal of the benzyl ester to give acid **17** was achieved in good yield via hydrogenation (Scheme 4).

Scheme 1[a]

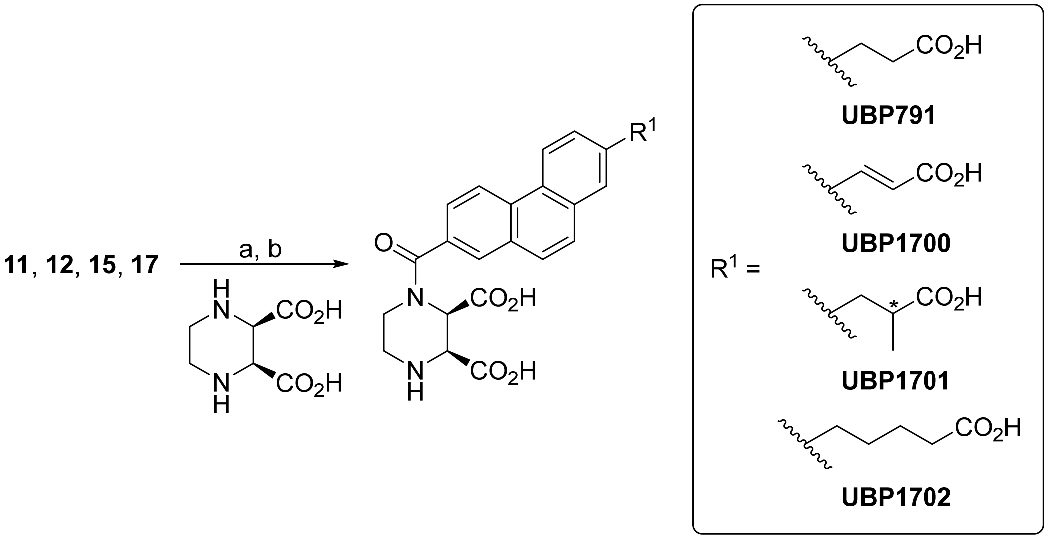

[a]Reagents and conditions: (a) (i) $SOCl_2$, reflux, 18 h, (ii) cis-piperazine-2,3-dicarboxylic acid, 1 M NaOH (aq), dioxane, 0 °C, 2 h then rt, 18 h, (iii) 2 M HCl (aq); (b) (i) LiOH, H_2O, rt, 24-36 h, (ii) AG50 resin.

Scheme 2[a]

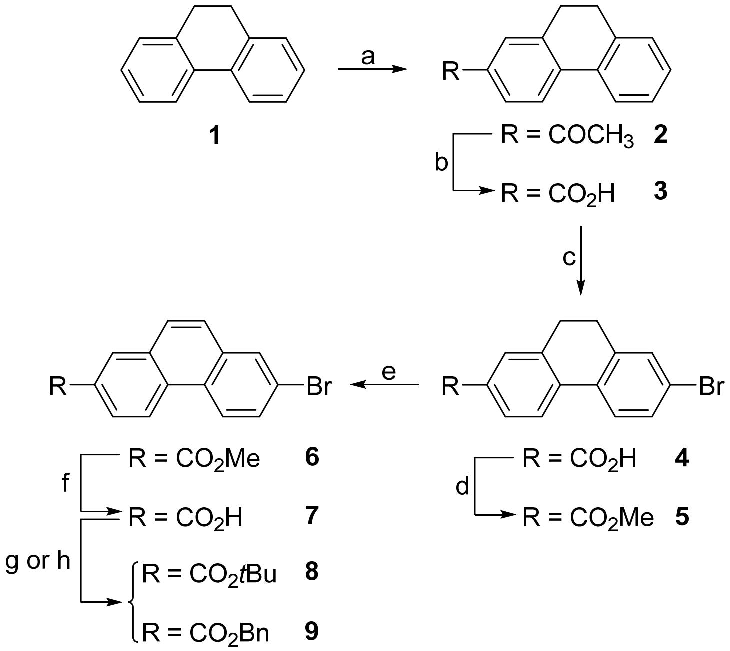

[a]Reagents and conditions: (a) (i) AcCl, $AlCl_3$, CS_2, 0 °C then reflux 4 h, (ii) conc HCl; (b) (i) NaOBr, dioxane, H_2O, 65 °C, 2 h, (ii) conc HCl; (c) Br_2, $(MeO)_3P(O)$, rt, 18 h; (d) MeOH, conc H_2SO_4, reflux 18 h; (e) (i) NBS, BPO, CCl_4, reflux 18 h, (ii) NaOAc, AcOH, reflux 1 h; (f) (i) LiOH, dioxane/H_2O, 65 °C, 3.5 h, (ii) 2 M HCl (aq); (g) (i) $SOCl_2$, reflux, 18 h, (ii) KO*t*Bu, THF, 0 °C, 1 h then rt, 18 h; (h) BnBr, K_2CO_3, DMF, rt, 18 h.

Scheme 3[a]

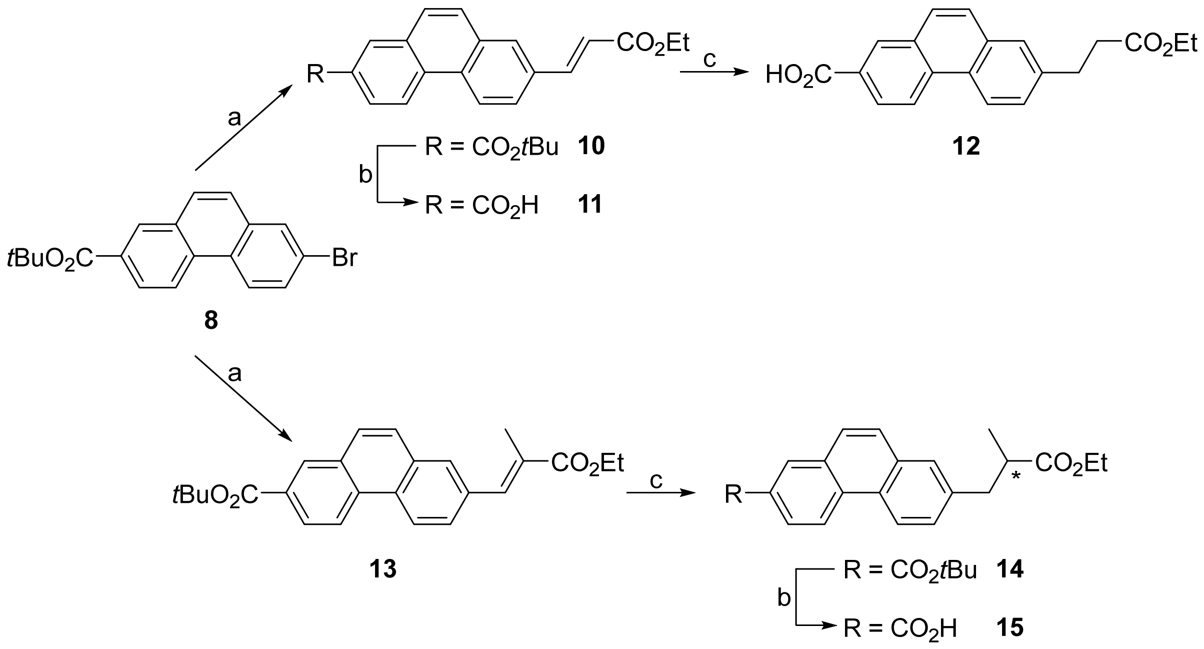

[a]Reagents and conditions: (a) Alkene, P(*o*-tolyl)$_3$, TEA, Pd(OAc)$_2$, DMF, 100 ^{o}C, 18 h; (b) TFA, 1,3-dimethoxybenzene, DCM; (c) 10% Pd/C, EtOAc, rt, 18 h.

Scheme 4[a]

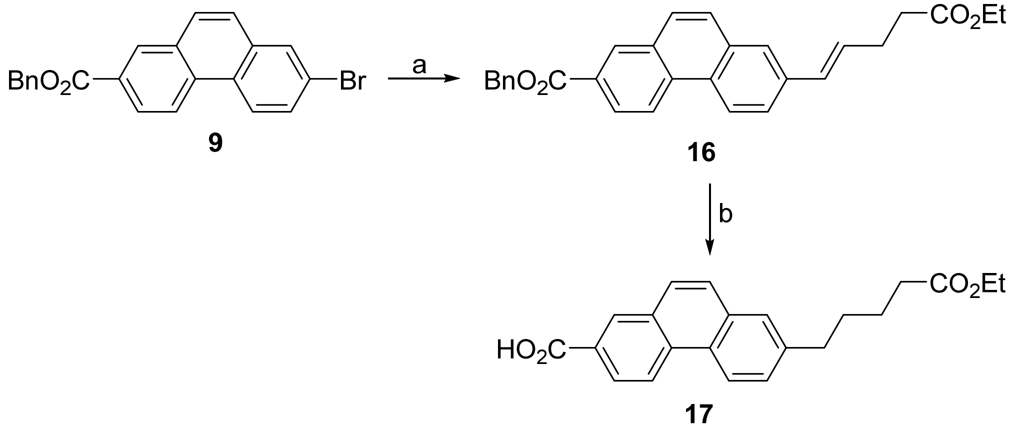

[a]Reagents and conditions: (a) Ethyl 4-pentenoate, P(*o*-tolyl)$_3$, TEA, Pd(OAc)$_2$, DMF, 100 ^{o}C, 18 h; (b) 10% Pd/C, 20% Pd(OH)$_2$/C, dioxane, rt, 18 h.

General Chemistry Procedures

Melting points were determined using a Mettler Toledo MP50 melting point system and are uncorrected. ^{1}H-NMR spectra were measured on a Bruker Ascend 400 spectrometer at 399.99 MHz or a Bruker Ascend 500 spectrometer with a DCH500S1 Cryo Probe at 500.23 MHz. ^{13}C-NMR spectra were recorded on a Bruker Ascend 500 spectrometer with a DCH500S1 Cryo Probe at 126.79 MHz. Chemical shifts (δ) are reported in parts per million (ppm) with 3-(trimethylsilyl)propionic-2,2,3,3-d_4 acid sodium salt in D_2O, or tetramethylsilane in $CDCl_3$ or DMSO-d_6 used as internal standards. Mass spectrometry was performed in the mass spectroscopy laboratories of the Department of Chemistry, University of Bristol, UK. Elemental analyses were performed by Elemental Microanalysis Ltd, Okehampton, UK. The purity of all novel compounds was determined by combustion analysis, which confirmed that there were ≥ 95% pure. Thin layer chromatography was performed on Merck silica gel 60 F_{254} plastic sheets. Flash chromatography was performed on Merck silica gel 60 (220-440 mesh) from Fisher. For thin layer chromatography of amino acids [2 (pyridine : acetic acid : water (3 : 8 : 11)) : 3 (*n*-butanol)] was utilised as the eluent. Amino acids were detected by spraying plates with a 2% solution of ninhydrin in 70% ethanol. All anhydrous reactions were conducted under argon. All anhydrous solvents were obtained from Sigma-Aldrich, UK. Cis-piperazine-2,3-dicarboxylic acid was synthesized as described previously.[1]

2-Acetyl-9,10-dihydrophenanthrene (2). To a stirring solution of **1** (12.50 g, 69.4 mmol) and acetyl chloride (4.93 mL, 69.4 mmol) in anhydrous carbon disulphide (250 mL) at 0 °C was added aluminium trichloride (13.9 g, 104 mmol) portionwise so as to maintain a temperature < 10 °C. After complete addition, the reaction was diluted with carbon disulphide (approx. 250 mL) as the mixture had become increasingly viscous. Cooling was then removed, and the

reaction heated at reflux for 4 h before being allowed to cool to room temperature and stirred overnight. The reaction was then concentrated in vacuo (CAUTION: carry out in fumehood) and the resultant residue dissolved in DCM (250 mL). Crushed ice was added followed by conc. HCl (10 mL). The organic layer was isolated, washed with aqueous 2 M HCl (100 mL), brine (100 mL), dried over $MgSO_4$ and concentrated in vacuo to yield a viscous orange oil. Purification via flash chromatography (10% EtOAc in hexane) afforded **2** as a viscous orange oil (12.40 g, 80%); ^{1}H NMR (400 MHz, $CDCl_3$) δ 2.63 (s, 3H), 2.87-2.98 (m, 4H), 7.26-7.37 (m, 3H), 7.78-7.85 (m, 3H), 7.89 (dd, J = 8.0 & 1.6 Hz, 1H); ^{13}C NMR (125 MHz, $CDCl_3$) δ 26.6, 28.8, 28.9, 123.8, 124.4, 127.2, 127.2, 128.0, 128.3, 128.6, 133.4, 135.8, 137.5, 138.0, 139.2, 197.9.

9,10-Dihydrophenanthrene-2-carboxylic acid (3). A stirring suspension of **2** (10.30 g, 46.3 mmol) in dioxane (100 mL) was heated at 65 °C until complete dissolution of the solid. At the same time, a solution of sodium hypobromite was prepared via the dropwise addition of bromine (11.9 mL, 231.5 mmol) to an ice-cooled aqueous solution of NaOH (32.4 g, 810.3 mmol dissolved in 150 mL of water). The sodium hypobromite solution was subsequently added dropwise to the dioxane solution over the course of 15 mins. After complete addition, the resultant mixture was stirred at 65 °C for 2 h before being allowed to cool to room temperature. The dioxane was removed in vacuo and the resultant aqueous solution topped up with water (50 mL), cooled to 0 °C, and acidified to pH 1 using conc. HCl. The solid which precipitated from solution was filtered off, washed thoroughly with water and allowed to air-dry on the sinter for 1.5 h. Further drying over P_2O_5 overnight afforded **3** as an off-white/light yellow solid (9.10 g, 88%) which was taken forward without further purification or characterisation.

7-Bromo-9,10-Dihydrophenanthrene-2-carboxylic acid (4). To a stirring solution of **3** (13.00 g, 58.0 mmol) in trimethyl phosphate (150 mL) was added dropwise bromine (6.2 mL, 121.8 mmol) dissolved in trimethyl phosphate (15 mL). After complete addition, the reaction was protected from light and stirred overnight at room temperature. The now suspension was placed in the fridge for 6 h before being poured onto ice. After the ice had melted the precipitate was filtered off and washed thoroughly with water until the filtrate ran clear. Drying over P_2O_5 yielded a pale-yellow solid which was re-crystallised from a 40% EtOH in toluene mixture to afford **4** as an off-white solid (8.10 g, 46%); mp: > 250 ºC (Lit.[2] 321-322 ºC); ^{1}H NMR (400 MHz, DMSO-d_6) δ 2.81-2.93 (m, 4H),7.52 (dd, J = 8.4 & 2.0 Hz, 1H), 7.54-7.57 (m, 1H), 7.80-7.90 (m, 3H), 7.93 (d, J = 8.0 Hz, 1H), 12.90 (br s, 1H); ^{13}C NMR (125 MHz, DMSO-d_6) δ 27.6, 27.7, 121.6, 123.9, 126.5, 128.2, 129.1, 129.8, 130.0, 130.9, 132.3, 137.0, 137.1, 140.3, 167.1.

Methyl 7-Bromo-9,10-dihydrophenanthrene-2-carboxylate (5). A stirring suspension of **4** (8.00 g, 26.4 mmol) in anhydrous methanol (300 mL) containing conc. H_2SO_4 (3 mL) was heated at reflux for 18 h. After cooling to room temperature, the reaction mixture was concentrated in vacuo and the resultant residue taken-up in DCM (150 mL) and washed with saturated $NaHCO_3$ (3 × 40 mL), water (50 mL) and brine (50 mL). After drying over MgSO4, concentration in vacuo afforded **5** as a light yellow solid (7.97 g, 95%); mp: 120-121 ºC (Lit.[2] 119-120 ºC); ^{1}H NMR (400 MHz, $CDCl_3$) δ 2.85-2.97 (m, 4H), 3.99 (s, 3H), 7.44 (d, *J* = 2.0 Hz, 1H), 7.48 (dd, *J* = 8.0 & 2.0 Hz, 1H), 7.66 (d, *J* = 8.4 Hz, 1H), 7.78 (d, *J* = 8.0 Hz, 1H), 7.94 (s, 1H), 7.99 (dd, *J* = 8.4 & 2.0 Hz, 1H); ^{13}C NMR (125 MHz, $CDCl_3$) δ 28.6, 52.1, 122.4, 123.5, 125.9, 128.4, 129.1, 129.4, 130.2, 131.2, 132.5, 137.0, 138.0, 140.0, 167.0.

Methyl 7-Bromophenanthrene-2-carboxylate (6). To a stirring solution of **5** (7.96 g, 25.1 mmol) in CCl_4 (175 mL) was added NBS (4.91 g, 27.6 mmol) followed by benzoyl peroxide (607.9 mg, 2.5 mmol). The resultant mixture was heated at reflux for 18 h. After this time, sodium acetate (8.0 g) and glacial acetic acid (8 mL) were added and heating at reflux continued for 1 h. The reaction was then allowed to cool to room temperature before being concentrated in vacuo. The resultant solid was broken up and suspended in water (150 mL) before being filtered off and washed thoroughly with cold water. After being allowed to air-dry for 1 h the crude product was dissolved in DCM (approx. 150 mL) and the organic phase isolated, dried over $MgSO_4$ and concentrated in vacuo to afford **6** as a light yellow solid (7.81 g, 99%); mp: 130-133 ºC; ^{1}H NMR (400 MHz, $CDCl_3$) δ 4.01 (s, 3H), 7.70 (d, *J* = 8.8 Hz, 1H), 7.77 (dd, *J* = 8.8 & 2.0 Hz, 1H), 7.84 (d, *J* = 8.8 Hz, 1H), 8.07 (d, *J* = 2.0 Hz, 1H), 8.27 (dd, *J* = 8.8 & 2.0 Hz, 1H), 8.56 (d, *J* = 8.8 Hz, 1H), 8.61 (d, *J* = 2.0 Hz, 1H), 8.67 (d, *J* = 8.8 Hz, 1H); ^{13}C NMR (125 MHz, $CDCl_3$) δ 52.5, 122.0, 123.0, 125.2, 126.8, 127.1, 128.5, 128.6, 128.7, 130.3, 131.0, 131.1, 131.5, 133.1, 134.4, 167.1; HRMS-ESI calcd for $C_{16}H_{11}BrO_2$ $[M + Na]^+$ 336.9835; found 336.9831.

7-Bromophenanthrene-2-carboxylic acid (7). To a stirring suspension of **6** (7.78 g, 24.7 mmol) in a mixture of dioxane (100 mL) and water (20 mL) was added an aqueous solution of LiOH (2.37 g, 98.8 mmol dissolved in 40 mL of water). After complete addition the mixture was heated at 65 °C until TLC indicated complete hydrolysis. The reaction was then allowed to cool to room temperature and the dioxane removed in vacuo. The resultant aqueous suspension was topped up with water (50 mL) and then acidified to pH 1 using aqueous 2 M HCl. Filtration yielded a solid which was washed thoroughly with water and allowed to air-dry on the sinter for approx. 1.5 h. Further drying over P_2O_5 afforded **7** as a white solid (7.37 g, 99%); mp: > 250 ºC; ^{1}H NMR (400 MHz, DMSO-d_6) δ 7.87 (dd, *J* = 8.8 & 2.0 Hz, 1H), 7.91 (d, *J* = 8.8 Hz, 1H), 8.06

(d, J = 8.8 Hz, 1H), 8.19 (dd, J = 8.8 & 2.0 Hz, 1H), 8.33 (d, J = 2.0 Hz, 1H), 8.64 (d, J = 2.0 Hz, 1H), 8.84 (d, J = 8.8 Hz, 1H), 8.93 (d, J = 8.8 Hz, 1H); ^{13}C NMR (125 MHz, DMSO-d$_6$) δ 121.2, 123.5, 126.0, 126.5, 126.9, 128.0, 128.6, 129.4, 129.9, 130.5, 130.5, 131.1, 132.1, 134.0, 167.2; HRMS-ESI calcd for $C_{15}H_9BrO_2$ [M - H]$^-$ 298.9713; found 298.9715.

***t*-Butyl 7-Bromophenanthrene-2-carboxylate (8).** A stirring suspension of **7** (7.23 g, 24.0 mmol) in thionyl chloride (120 mL) containing a few drops of DMF as catalyst was heated at reflux for 18 h. The solvent was then carefully removed via distillation and the resultant residue azeotroped with anhydrous toluene (2 × 40 mL) to remove any remaining thionyl chloride. The crude acid chloride was dissolved in anhydrous THF (50 mL) and added dropwise to a stirring 0 °C solution of potassium *t*-butoxide (1.0 M soln in THF, 24.0 mL, 24.0 mmol) in anhydrous THF (75 mL). After complete addition, the reaction mixture was stirred at 0 °C for 1 h and then at room temperature for 18 h. The mixture was then concentrated in vacuo and the resultant residue partitioned between EtOAc (150 mL) and H_2O (100 mL). The organic layer was subsequently isolated, and the aqueous phase extracted with EtOAc (2 × 50 mL). The organic layers were combined, washed with H_2O (3 × 100 mL), brine (100 mL), dried over $MgSO_4$ and concentrated in vacuo to afford an orange oil which partially solidified on standing. To remove any inorganics still present, the crude product was dissolved /suspended in DCM (100 mL) and filtered through celite. The filter cake was washed thoroughly with DCM and the filtrate subsequently concentrated in vacuo to afford **8** as a viscous orange oil which partially solidified on standing (7.55 g, 88%); ^{1}H NMR (400 MHz, $CDCl_3$) δ 1.67 (s, 9H), 7.69 (d, J = 8.8 Hz, 1H), 7.77 (dd, J = 8.8 & 2.0 Hz, 1H), 7.84 (d, J =8.8 Hz, 1H), 8.07 (d, J = 2.0 Hz, 1H), 8.22 (dd, J = 8.8 & 2.0 Hz, 1H), 8.54 (d, J = 2.0 Hz, 1H), 8.56 (d, J = 8.8 Hz, 1H), 8.65 (d, J = 8.8 Hz, 1H); ^{13}C NMR (125 MHz, $CDCl_3$) δ 28.4, 81.6, 121.8, 122.7, 125.2, 126.6, 127.1, 128.6, 128.8, 130.2, 130.4, 130.8,

131.0, 131.4, 132.8, 134.4, 165.8; HRMS-ESI calcd for $C_{19}H_{17}BrO_2$ $[M + Na]^+$ 379.0304; found 379.0301.

Benzyl 7-Bromophenanthrene-2-carboxylate (9). To a stirring solution of **7** (2.05 g, 6.8 mmol) in anhydrous DMF (120 mL) was added K_2CO_3 (1.13 g, 8.2 mmol). The resultant suspension was stirred rapidly at room temperature for 1 h before benzyl bromide (0.97 mL, 8.2 mmol) was added. The reaction was then stirred at room temperature for 18 h after which the solvent was removed in vacuo and the resultant residue partitioned between EtOAc (50 mL) and H_2O (50 mL). The organic layer was isolated, and the aqueous phase extracted with EtOAc (2 × 25 mL). The organic layers were pooled, washed with H_2O (5 × 50 mL), brine (50 mL), dried over $MgSO_4$ and concentrated in vacuo to afford **9** as a white solid (2.08 g, 78%); mp: 169-172 ºC; ^1H NMR (400 MHz, $CDCl_3$) δ 5.46 (s, 2H), 7.40-7.46 (m, 3H), 7.49-7.54 (m, 2H), 7.68 (d, *J* = 8.8 Hz, 1H), 7.76 (dd, *J* = 8.8 & 2.4 Hz, 1H), 7.82 (d, *J* = 8.8 Hz, 1H), 8.06 (d, *J* = 2.0 Hz, 1H), 8.29 (d, *J* = 8.8 & 2.0 Hz, 1H), 8.54 (d, *J* = 8.8 Hz, 1H), 8.63 (d, *J* = 2.0 Hz, 1H), 8.65 (d, *J* = 8.8 Hz); ^{13}C NMR (125 MHz, $CDCl_3$) δ67.0, 121.9, 122.8, 125.0, 126.6, 127.0, 128.3, 128.3, 128.4, 128.5, 128.6, 128.7, 130.1, 130.8, 131.1, 131.3, 133.0, 134.3, 136.0, 166.3; HRMS-ESI calcd for $C_{22}H_{15}BrO_2$ $[M + Na]^+$ 413.0148; found, 413.0149.

***t*-Butyl (*E*)-7-(3-Ethoxy-3-oxoprop-1-en-1-yl)phenanthrene-2-carboxylate (10).** A flask was charged with **8** (4.00 g, 11.2 mmol), palladium acetate (25.1 mg, 1 mol%) and tri-*o*-tolylphosphine (136 mg, 4 mol%). The flask was then briefly evacuated and backfilled with argon three times. Degassed anhydrous DMF (40 mL) was added followed by ethyl acrylate (1.83 mL, 16.8 mmol) and triethylamine (2.34 mL, 16.8 mmol). The resultant mixture was heated at 100 °C overnight. After being allowed to cool to room temperature the reaction mixture

was filtered through a celite pad to remove any precipitated Pd(0) and then poured into a stirred solution of EtOAc (100 mL), water (100 mL) and aqueous 1 M HCl (10 mL). The organic layer was subsequently isolated and the aqueous phase further extracted with EtOAc (2 × 50 mL). The organic extracts were pooled, washed with water (5 × 100 mL), brine (100 mL) and dried over $MgSO_4$. Concentration in vacuo afforded an orange oil which was purified by flash chromatography (DCM) to afford **10** as a viscous yellow oil (2.51 g, 59%); ^{1}H NMR (400 MHz, $CDCl_3$) δ 1.36 (t, *J* = 7.2 Hz, 3H), 1.66 (s, 9H), 4.30 (q, *J* = 7.2 Hz, 2H), 6.61 (d, *J* = 16.0 Hz, 1H), 7.77 (d, *J* = 9.2 Hz, 1H), 7.81-7.90 (m, 3H), 8.00 (d, *J* = 1.6 Hz, 1H), 8.21 (dd, *J* = 8.4 & 1.6 Hz, 1H), 8.53 (d, *J* = 1.6 Hz, 1H), 8.67 (d, *J* = 6.0 Hz, 1H), 8.69 (d, *J* = 6.0 Hz, 1H); ^{13}C NMR (125 MHz, $CDCl_3$) δ 14.5, 28.4, 60.8, 81.6, 119.4, 123.1, 124.2, 125.3, 127.0, 127.7, 128.3, 129.7, 130.6, 130.8, 131.1, 132.0, 132.7, 133.1, 133.6, 144.2, 165.8, 167.1; HRMS-ESI calcd for $C_{24}H_{24}O_4$ $[M + Na]^+$ 399.1657; found 399.1650.

(*E*)-7-(3-Ethoxy-3-oxoprop-1-en-1-yl)phenanthrene-2-carboxylic acid (11). To a stirring solution of **10** (2.48 g, 6.6 mmol) and 1,3-dimethoxybenzene (1.73 mL, 13.2 mmol) in anhydrous DCM (20 mL) was added carefully TFA (7 mL). The resultant mixture was stirred at room temperature until TLC indicated complete deprotection. After 18 h, the now suspension was filtered to afford **11** as a pale-yellow solid which was washed thoroughly with Et_2O and allowed to air dry. The filtrate was concentrated in vacuo and the resultant residue azeotroped with anhydrous toluene (2 × 15 mL) to try and remove any remaining TFA. Et_2O (20 mL) was added and the resultant suspension filtered to afford additional **11** which was washed thoroughly with Et_2O and then allowed to air dry (1.69 g, 80%); mp: >250 ºC; ^{1}H NMR (400 MHz, DMSO-d_6) δ 1.29 (t, *J* = 7.2 Hz, 3H), 4.23 (q, *J* = 7.2 Hz, 2H), 6.87 (d, *J* = 16.0 Hz, 1H), 7.86 (d, *J* = 16.0 Hz, 1H), 7.93 (d, *J* = 8.8 Hz, 1H), 8.05 (d, *J* = 8.8 Hz, 1H), 8.11 (dd, *J* = 8.4 & 1.2 Hz, 1H), 8.18 (dd,

J = 8.4 & 1.2 Hz, 1H), 8.37 (d, J = 1.2 Hz, 1H), 8.63 (d, J = 1.2 Hz, 1H), 8.90 (d, J = 8.8 Hz, 1H), 8.96 (d, J = 8.8 Hz, 1H); ^{13}C NMR (125 MHz, DMSO-d$_6$) δ 14.3, 60.2, 119.4, 123.8, 124.4, 125.9, 126.8, 127.7, 128.0, 129.4, 129.7, 130.4, 130.5, 131.6, 132.2, 132.6, 133.4, 143.8, 166.2, 167.3; HRMS-ESI calcd for $C_{20}H_{16}O_4$ [M - H]$^-$ 319.3365; found 319.3360.

7-(3-Ethoxy-3-oxopropyl)phenanthrene-2-carboxylic acid (12). A solution of **11** (2.79 g, 6.8 mmol) in EtOAc (100 mL) was hydrogenated under 3 bar of hydrogen in the presence of 10wt% palladium on activated carbon (75 mg) for 18 h. The reaction mixture was then filtered through a Celite pad before being concentrated in vacuo to afford **12** as an off-white solid (2.15 g, 98%); mp: >250 °C; ^{1}H NMR (400 MHz, DMSO-d$_6$) δ 1.15 (t, J = 7.2 Hz, 3H), 2.77 (t, J = 7.6 Hz, 2H), 3.09 (t, J = 7.2 Hz, 2H), 4.05 (q, J = 7.2 Hz, 2H), 7.64 (dd, J = 8.8 & 2.0, 1H), 7.85 (m, 1H), 7.86 (d, J = 8.8 Hz, 1H), 7.97 (d, J = 8.8 Hz, 1H), 8.15 (dd, J = 8.8 & 2.0, 1H), 8.60 (d, J = 2.0, 1H), 8.79 (d, J = 8.8 Hz, 1H), 8.89 (d, J = 8.8 Hz, 1H); ^{13}C NMR (100 MHz, DMSO-d$_6$) δ 14.5, 30.2, 34.7, 59.9, 123.2, 123.7, 126.5, 127.28, 127.45, 127.5, 127.6, 128.0, 130.3, 130.8, 132.4, 132.5, 140.3, 167.4, 172.6; HRMS-ESI calcd for $C_{20}H_{18}O_4$ [M + Na]$^+$ 321.1132; found 321.1129.

***t*-Butyl (*E*)-7-(3-Ethoxy-2-methyl-3-oxoprop-1-en-1-yl)phenanthrene-2-carboxylate (13).** Method identical to that described for **10**. **8** (1.86 g, 5.2 mmol), palladium acetate (11.7 mg, 1 mol%), tri-*o*-tolylphosphine (63.3 mg, 4 mol%), ethyl methacrylate (0.97 mL, 7.8 mmol), triethylamine (1.09 mL, 7.8 mmol) and anhydrous DMF (40 mL) afforded a dark orange oil which was purified by flash chromatography (DCM) to afford **13** as a viscous yellow oil which partially solidified on standing (1.22 g, 60%); ^{1}H NMR (500 MHz, $CDCl_3$) δ 1.41 (t, J = 7.0 Hz, 3H), 1.70 (s, 9H), 2.27 (d, J = 6.5 Hz, 3H), 4.35 (q, J = 7.0 Hz, 2H), 7.75 (dd, J = 8.5 & 2.0 Hz, 1H), 7.81 (d, J = 9.0 Hz, 1H), 7.86 (d, J = 9.0 Hz, 1H), 7.91 (s, 1H), 7.96 (d, J = 2.0 Hz, 1H),

8.25 (dd, *J* = 8.5 & 1.5 Hz, 1H), 8.57 (d, *J* = 2.0 Hz, 1H), 8.72 (d, *J* = 8.5 Hz, 1H), 8.73 (d, *J* = 8.5 Hz, 1H); ^{13}C NMR (125 MHz, $CDCl_3$) δ 14.3, 14.4, 28.3, 61.0, 81.4, 122.8, 123.4, 126.8, 127.6, 127.9, 128.1, 129.4, 129.6, 129.8, 130.2, 130.6, 131.7, 132.7, 135.1, 138.1, 165.7, 168.6; HRMS-ESI calcd for $C_{25}H_{26}O_4$ $[M + Na]^+$ 413.1723; found 413.1731.

***t*-Butyl (*RS*)-7-(3-Ethoxy-2-methyl-3-oxopropyl)phenanthrene-2-carboxylate (14).** A solution of **13** (1.21 g, 3.1 mmol) in EtOAc (100 mL) was hydrogenated under 3 bar of hydrogen in the presence of 10 wt % palladium on activated carbon (50 mg) for 18 h. The reaction mixture was then filtered through a Celite pad before being concentrated in vacuo to afford **14** as a pale-yellow oil (1.19 g, 98%); ^{1}H NMR (400 MHz, $CDCl_3$) δ 1.17 (t, *J* = 7.2 Hz, 3H), 1.67 (s, 9H), 1.22 (d, *J* = 6.8 Hz, 3H), 2.83-2.93 (m, 2H), 3.20-3.29 (m, 1H), 4.10 (q, *J* = 7.2 Hz, 2H), 7.52 (dd, *J* = 8.8 & 1.6 Hz, 1H), 7.70 (d, *J* = 1.6 Hz, 1H), 7.72 (d, *J* = 8.8 Hz, 1H), 7.79 (d, *J* = 8.8 Hz, 1H), 8.19 (dd, *J* = 8.8 & 1.6 Hz, 1H), 8.53 (d, *J* = 1.6 Hz, 1H), 8.62 (d, *J* = 8.8 Hz, 1H), 8.66 (d, *J* = 8.8 Hz, 1H); ^{13}C NMR (125 MHz, $CDCl_3$) δ 14.2, 17.0, 28.3, 39.7, 41.5, 60.4, 81.2, 122.5, 123.4, 126.5, 127.4, 127.4, 128.2, 128.3, 128.5, 129.6, 130.5, 131.2, 132.9, 133.0, 139.0, 165.9, 176.0; HRMS-ESI calcd for $C_{25}H_{28}O_4$ $[M + Na]^+$ 415.1880; found 415.1873.

(*RS*)-7-(3-Ethoxy-2-methyl-3-oxopropyl)phenanthrene-2-carboxylic acid (15). Method identical to that described for **11**. **14** (1.14 g, 2.9 mmol), 1,3-dimethoxybenzene (0.76 mL, 5.8 mmol), TFA (6 mL) and anhydrous DCM (10 mL) afforded **15** as a gummy white solid (926 mg, 95%) which was taken forward without further purification or characterisation.

Benzyl (*E*)-7-(5-Ethoxy-5-oxopent-1-en-1-yl)phenanthrene-2-carboxylate (16). Method identical to that described for **10**. **9** (3.00 g, 7.7 mmol), palladium acetate (17.2 mg, 1 mol%),

tri-*o*-tolylphosphine (93.4 mg, 4 mol%), ethyl 4-pentenoate (1.64 mL, 11.5 mmol), triethylamine (1.60 mL, 11.5 mmol) and anhydrous DMF (40 mL) afforded a dark orange oil which was purified by flash chromatography (DCM) to afford **16** as a yellow solid (2.13 g, 63%); mp: 150-152 ºC; ^{1}H NMR (400 MHz, $CDCl_3$) δ 1.28 (t, *J* = 7.2 Hz, 3H), 2.52-2.57 (m, 2H), 2.62 (t, *J* = 6.8 Hz, 2H), 4.17 (q, *J* = 7.2 Hz, 2H), 5.45 (s, 2H), 6.44 (dt, *J* = 16.0 & 6.8 Hz, 1H), 6.64 (d, *J* = 16.0 Hz, 1H), 7.34-7.46 (m, 4H), 7.49-7.55 (m, 2H), 7.71-7.83 (m, 3H), 8.27 (dd, *J* = 8.8 & 2.0 Hz, 1H), 8.59-8.64 (m, 2H), 8.67 (d, *J* = 8.8 Hz, 1H); ^{13}C NMR (125 MHz, $CDCl_3$) δ 14.3, 28.5, 34.0, 60.5, 66.9, 122.9, 123.6, 124.7, 126.2, 126.6, 127.5, 127.7, 127.8, 128.3, 128.3, 128.7, 128.8, 130.1, 130.6, 131.0, 131.3, 133.2, 133.4, 136.1, 136.8, 166.5, 173.0; HRMS-ESI calcd for $C_{29}H_{26}O_4$ $[M + Na]^+$ 461.1723; found 461.1730.

7-(5-Ethoxy-5-oxopentyl)phenanthrene-2-carboxylic acid (17). A solution of **16** (1.97 g, 4.5 mmol) in dioxane (100 mL) was hydrogenated under 3 bar of hydrogen in the presence of 10wt% palladium on carbon (296 mg, 15 w/w%) and 20 wt% palladium hydroxide on carbon (296 mg, 15 w/w%) for 18 h. The reaction mixture was then filtered through a Celite pad before being concentrated in vacuo to afford **17** (1.50 g, 95%) as a viscous clear oil which was utilised without further purification or characterisation.

(2*R,3*S**)-1-(7-(2-carboxyethyl)phenanthrene-2-carbonyl)piperazine-2,3-dicarboxylic acid (UBP791).** A stirring suspension of **12** (2.13 g, 6.6 mmol) in thionyl chloride (50 mL) containing a few drops of DMF as catalyst was heated at reflux for 18 h. The solvent was then carefully removed via distillation and the resultant residue azeotroped with anhydrous toluene (3 × 20 mL) to remove any remaining thionyl chloride. The crude acid chloride was dissolved in anhydrous dioxane (25 mL) and added dropwise to a rapidly stirring 0 °C solution of cis-piperazine-2,3-

dicarboxylic acid (1.27 g, 6.6 mmol) dissolved in a mixture of aqueous 1 M NaOH (19.8 mL, 19.8 mmol) and dioxane (60 mL). The resultant mixture was stirred at 0 °C for 2 h before being allowed to warm to room temperature and stirred for a further 18 h. The pH was then adjusted to 7 using aqueous 2 M HCl and the dioxane removed in vacuo. The resultant aqueous solution was topped up with water (50 mL) and acidified to pH 3 using aqueous 2 M HCl. The solid which precipitated from solution was filtered off and washed with water (3 × 40 mL) before being allowed to air-dry on the sinter for 2 h. Further drying over P_2O_5 overnight afforded the crude ester as a light brown solid (2.67 g, 84%) which was utilised without further purification or characterisation.

An aqueous solution of LiOH (776 mg, 32.4 mmol, dissolved in 30 mL of water) was added dropwise to a stirring suspension of the crude ester (2.58 g, 5.4 mmol) in a mixture of dioxane (60 mL) and water (20 mL). The resultant mixture was stirred rapidly at room temperature until TLC indicated complete hydrolysis. After 24 h, the pH was adjusted to 7 using aqueous 2 M HCl and the dioxane removed in vacuo. The resultant aqueous solution was topped up with water (50 mL) and Dowex 50WX8-400 ion exchange resin (H^+ form) (0.25 mmol of cation/mL of resin; 152 mL) added. The mixture was stirred carefully for 30 mins before being poured onto an equivalent volume of identical resin. The column was then eluted with water until no ninhydrin positive fractions were observed, a 1:1 dioxane/water mix until no UV fractions were observed, and finally aqueous 1 M pyridine. Those pyridine fractions which were ninhydrin positive were pooled and concentrated in vacuo to yield a white solid which was azeotroped with water (4 × 25 mL) to ensure complete removal of any remaining pyridine. The solid was subsequently broken up, suspended in diethyl ether (50 mL) and filtered off to afford UBP791 as an off-white solid (171 mg, 7%); mp: >250 ºC; ^{1}H NMR (400 MHz, D_2O/NaOD, pH 11) δ 2.35-2.55 (m, 2H), 2.55-3.20 (m, 5H), 3.25 (d, J = 3.5 Hz, 0.5H), 3.31 (d, J = 3.5 Hz, 0.5H), 3.48 (d, J

=13.5 Hz, 0.5H), 4.29 (td, *J* = 13.5 & 2.0 Hz, 0.5H), 4.79 (usp, 0.5H), 5.46-5.49 (m, 0.5H), 7.04-7.72 (m, 5H), 7.86-7.97 (m, 1H), 8.39-8.65 (m, 2H); HRMS-ESI calcd for $C_{24}H_{22}N_2O_7$ $[M - H]^-$ 449.1349; found 449.1358; Found: C, 59.81; H, 5.34; N, 5.80. Calc. for $C_{25}H_{24}N_2O_7 \cdot 1.61H_2O$: C, 60.12; H, 5.30; N, 5.84%.

(2*R,3*S**)-1-(7-((*E*)-2-Carboxyvinyl)phenanthrene-2-carbonyl)piperazine-2,3-dicarboxylic acid (UBP1700).** A stirring suspension of **11** (1.60 g, 5.0 mmol) in thionyl chloride (40 mL) containing a few drops of DMF as catalyst was heated at reflux for 18 h. The solvent was then carefully removed via distillation and the resultant residue azeotroped with anhydrous toluene (3 × 15 mL) to remove any remaining thionyl chloride. The crude acid chloride was dissolved in anhydrous dioxane (30 mL) and added dropwise to a rapidly stirring 0 °C solution of cis-piperazine-2,3-dicarboxylic acid (961 mg, 5.0 mmol) dissolved in a mixture of aqueous 1 M NaOH (15.0 mL, 15.0 mmol) and dioxane (30 mL). The resultant mixture was stirred at 0 °C for 2 h before being allowed to warm to room temperature and stirred for a further 18 h. The pH was then adjusted to 7 using aqueous 2 M HCl and the dioxane removed in vacuo. The resultant aqueous solution was topped up with water (50 mL) and acidified to pH 3 using aqueous 2 M HCl. The solid which precipitated from solution was filtered off, washed with water (3 × 40 mL), hot dioxane (3 × 40 mL) and then Et_2O (40 mL) to afford the crude ester as a pale-yellow solid (1.54 g, 65%) which was utilised without further purification or characterisation.

An aqueous solution of LiOH (302 mg, 12.6 mmol, dissolved in 10 mL of water) was added dropwise to a stirring suspension of the crude ester (1.00 g, 2.1 mmol) in water (40 mL). The resultant mixture was stirred rapidly at room temperature until TLC indicated complete hydrolysis. After 24 h, the pH was adjusted to 7 using aqueous 2 M HCl and Dowex 50WX8-400 ion exchange resin (H^+ form) (0.25 mmol of cation/mL of resin; 59 mL) added. The mixture was

stirred carefully for 30 mins before being poured onto an equivalent volume of identical resin. The column was then eluted with water until no ninhydrin positive fractions were observed, a 1:1 dioxane/water mix until no UV fractions were observed, and finally aqueous 1 M pyridine. Those pyridine fractions which were ninhydrin and $KMnO_4$ positive were pooled and concentrated in vacuo to yield an off-white solid which was azeotroped with water (4 × 20 mL) to ensure complete removal of any remaining pyridine. The solid was subsequently broken up, suspended in diethyl ether (25 mL) and filtered off to afford UBP1700 as an off-white solid (142 mg, 15%); mp: >250 ºC; ^{1}H NMR (400 MHz, D_2O/NaOD, pH 11) δ 2.55-3.23 (m, 3H), 3.31 (d, *J* = 3.6 Hz, 0.5H), 3.40 (d, *J* = 3.6 Hz, 0.5H), 3.61 (d, *J* = 13.9 Hz, 0.5H), 4.40 (d, *J* = 13.9 Hz, 0.5H), 4.79 (usp, 0.5H), 5.63 (d, *J* = 3.6 Hz, 0.5H), 6.90 (m, 1H), 7.63-8.12 (m, 7H), 8.58-8.79 (m, 2H); HRMS-MALDI calcd for $C_{24}H_{20}N_2O_7$ $[M - H]^-$ 447.1198; found 447.1205; Found: C, 56.27; H, 4.90; N, 5.82. Calc. for $C_{24}H_{20}N_2O_7 \cdot 3.4H_2O$: C, 56.56; H, 5.30; N, 5.50%.

(2*R,3*S**)-1-(7-((*RS*)-2-Carboxypropyl)phenanthrene-2-carbonyl)piperazine-2,3-dicarboxylic acid (UBP1701).** A stirring suspension of **15** (1.01 g, 3.0 mmol) in thionyl chloride (40 mL) containing a few drops of DMF as catalyst was heated at reflux for 18 h. The solvent was then carefully removed via distillation and the resultant residue azeotroped with anhydrous toluene (3 × 15 mL) to remove any remaining thionyl chloride. The crude acid chloride was dissolved in anhydrous dioxane (25 mL) and added dropwise to a rapidly stirring 0 °C solution of cis-piperazine-2,3-dicarboxylic acid (576 mg, 3.0 mmol) dissolved in a mixture of aqueous 1 M NaOH (9.0 mL, 9.0 mmol) and dioxane (30 mL). The resultant mixture was stirred at 0 °C for 2 h before being allowed to warm to room temperature and stirred for a further 18 h. The pH was then adjusted to 7 using aqueous 2 M HCl and the dioxane removed in vacuo. The resultant aqueous solution was topped up with water (50 mL) and acidified to pH 3 using aqueous 2 M

HCl. The solid which precipitated from solution was filtered off, and washed with water (3 × 40 mL) before being allowed to air-dry for 1.5 h. Further drying over P_2O_5 overnight afforded the crude ester as a light brown solid (1.21 g, 82%) which was utilised without further purification or characterisation.

An aqueous solution of LiOH (287 mg, 12.0 mmol, dissolved in 10 mL of water) was added dropwise to a stirring suspension of the crude ester (985 mg, 2.0 mmol) in a mixture of dioxane (40 mL) and water (60 mL). The resultant mixture was stirred rapidly at room temperature until TLC indicated complete hydrolysis. After 36 h, the pH was adjusted to 7 using aqueous 2 M HCl and the dioxane removed in vacuo. The resultant aqueous solution was topped up with water (40 mL) and Dowex 50WX8-400 ion exchange resin (H^+ form) (0.25 mmol of cation/mL of resin; 56 mL) added. The mixture was stirred carefully for 30 mins before being poured onto an equivalent volume of identical resin. The column was then eluted with water until no ninhydrin positive fractions were observed, a 1:1 dioxane/water mix until no UV fractions were observed, and finally aqueous 1 M pyridine. Those pyridine fractions which were ninhydrin positive were pooled and concentrated in vacuo to yield a white solid which was azeotroped with water (4 × 25 mL) to ensure complete removal of any remaining pyridine. The solid was subsequently broken up, suspended in diethyl ether (25 mL) and filtered off to afford UBP1701 as an off-white solid (148 mg, 16%); mp: >250 ºC; ^{1}H NMR (400 MHz, D_2O/NaOD, pH 11) δ 1.20-1.23 (m, 3H), 2.55-3.30 (m, 6H), 3.35 (d, *J* = 3.6 Hz, 0.5H), 3.48 (d, *J* = 3.6 Hz, 0.5H), 3.63 (d, *J* = 13.9 Hz, 0.5H), 4.33 (d, *J* = 13.9 Hz, 0.5H), 4.83 (d, J = 3.6 Hz, 0.5H), 5.59 (d, *J* = 3.6 Hz, 0.5H), 7.10-7.79 (m, 6H), 7.95-8.12 (m, 2H); HRMS-ESI calcd for $C_{25}H_{24}N_2O_7$ $[M - H]^-$ 463.1505; found 463.1504; Found: C, 58.57; H, 5.59; N, 5.95. Calc. for $C_{25}H_{24}N_2O_7 \cdot 2.44H_2O$: C, 58.95; H, 5.74; N, 5.50%.

(2*R,3*S**)-1-(7-(4-Carboxybutyl)phenanthrene-2-carbonyl)piperazine-2,3-dicarboxylic acid (UBP1702).** A stirring suspension of **17** (1.61 g, 4.6 mmol) in thionyl chloride (50 mL) containing a few drops of DMF as catalyst was heated at reflux for 18 h. The solvent was then carefully removed via distillation and the resultant residue azeotroped with anhydrous toluene (3 × 25 mL) to remove any remaining thionyl chloride. The crude acid chloride was dissolved in anhydrous dioxane (25 mL) and added dropwise to a rapidly stirring 0 °C solution of cis-piperazine-2,3-dicarboxylic acid (884 mg, 4.6 mmol) dissolved in a mixture of aqueous 1 M NaOH (13.8 mL, 13.8 mmol) and dioxane (40 mL). The resultant mixture was stirred at 0 °C for 2 h before being allowed to warm to room temperature and stirred for a further 18 h. The pH was then adjusted to 7 using aqueous 2 M HCl and the dioxane removed in vacuo. The resultant aqueous solution was topped up with water (50 mL) and acidified to pH 3 using aqueous 2 M HCl. The solid which precipitated from solution was filtered off, and washed with water (3 × 40 mL) before being allowed to air-dry for 1.5 h. Further drying over P_2O_5 overnight afforded the crude ester as a light brown solid (1.35 g, 58%) which was utilised without further purification or characterisation.

An aqueous solution of LiOH (374 mg, 15.6 mmol, dissolved in 10 mL of water) was added dropwise to a stirring suspension of the crude ester (1.32 g, 2.6 mmol) in a mixture of dioxane (10 mL) and water (50 mL). The resultant mixture was stirred rapidly at room temperature until TLC indicated complete hydrolysis. After 24 h, the pH was adjusted to 7 using aqueous 2 M HCl and the dioxane removed in vacuo. The resultant aqueous solution was topped up with water (40 mL) and Dowex 50WX8-400 ion exchange resin (H^+ form) (0.25 mmol of cation/mL of resin; 73 mL) added. The mixture was stirred carefully for 30 mins before being poured onto an equivalent volume of identical resin. The column was then eluted with water until no ninhydrin positive fractions were observed, a 1:1 dioxane/water mix until no UV fractions

were observed, and finally aqueous 1 M pyridine. Those pyridine fractions which were ninhydrin positive were pooled and concentrated in vacuo to yield a white solid which was azeotroped with water (4 × 25 mL) to ensure complete removal of any remaining pyridine. The solid was subsequently broken up, suspended in diethyl ether (25 mL) and filtered off to afford UBP1702 as a tan solid (102 mg, 8%); mp: >250 ºC; ^{1}H NMR (400 MHz, D_2O/NaOD, pH 11) δ 2.60-3.61 (12.5H), 4.33 (d, *J* = 13.9 Hz, 0.5H), 4.83 (d, J = 3.6 Hz, 0.5H), 5.59 (d, *J* = 3.6 Hz, 0.5H), 7.10-7.79 (m, 6H), 7.95-8.12 (m, 2H); HRMS-ESI calcd for $C_{26}H_{26}N_2O_7$ [M - H]$^-$ 477.1662; found 477.1674; Found: C, 58.29; H, 5.78; N, 5.11. Calc. for $C_{26}H_{26}N_2O_7 \cdot 3.0H_2O$: C, 58.64; H, 6.06; N, 5.26%.

Supplementray References

1 Buller, A. L. & Monaghan, D. T. Pharmacological heterogeneity of NMDA receptors: characterization of NR1a/NR2D heteromers expressed in Xenopus oocytes. *Eur J Pharmacol* **320**, 87-94, doi:10.1016/s0014-2999(96)00880-1 (1997).

2 Buller, A. L. *et al.* The molecular basis of NMDA receptor subtypes: native receptor diversity is predicted by subunit composition. *J Neurosci* **14**, 5471-5484 (1994).

3 Erreger, K. *et al.* Subunit-specific agonist activity at NR2A-, NR2B-, NR2C-, and NR2D-containing N-methyl-D-aspartate glutamate receptors. *Mol Pharmacol* **72**, 907-920, doi:10.1124/mol.107.037333 (2007).

4 Jespersen, A., Tajima, N., Fernandez-Cuervo, G., Garnier-Amblard, E. C. & Furukawa, H. Structural insights into competitive antagonism in NMDA receptors. *Neuron* **81**, 366-378, doi:10.1016/j.neuron.2013.11.033 (2014).

5 Morley, R. M., Tse, H.-W., Miller, J. C., Monaghan, D. T. & Jane, D. E. Synthesis and pharmacology of N1-substituted piperazine-2,3-dicarboxylic acid derivatives acting as NMDA receptor antagonists. *J. Med. Chem.* **48**, 2627-2637 (2005).

6 Abdell, A. D., Brandt, M., Levy, M. A. & Holt, D. A. Preparation and biological activity of tricyclic non-steroidal inhibitors of human steroid 5α-reductase. *J. Chem. Soc. Perkin Trans. 1*, 1663-1668 (1997).
