# Supplementary material for: Structural basis of subtype-selective competitive antagonism for GluN2C/2D-containing NMDA receptors
Source: Nat Commun. 2020 Jan 22;11:423. doi: 10.1038/s41467-020-14321-0 (PMC6976569; doi:10.1038/s41467-020-14321-0)
Supplement: Supplementary file 2 — Reporting Summary [file 41467_2020_14321_MOESM2_ESM.pdf]

nature research

Corresponding author(s): Hiro Furukawa

Last updated by author(s): Dec 12, 2019

Reporting Summary

Nature Research wishes to improve the reproducibility of the work that we publish. This form provides structure for consistency and transparency in reporting. For further information on Nature Research policies, see Authors & Referees and the Editorial Policy Checklist.

Statistics

For all statistical analyses, confirm that the following items are present in the figure legend, table legend, main text, or Methods section.

n/a	Confirmed	
☐	☒	The exact sample size (n) for each experimental group/condition, given as a discrete number and unit of measurement
☐	☒	A statement on whether measurements were taken from distinct samples or whether the same sample was measured repeatedly
☐	☒	The statistical test(s) used AND whether they are one- or two-sided<br>*Only common tests should be described solely by name; describe more complex techniques in the Methods section.*
☐	☒	A description of all covariates tested
☐	☒	A description of any assumptions or corrections, such as tests of normality and adjustment for multiple comparisons
☐	☒	A full description of the statistical parameters including central tendency (e.g. means) or other basic estimates (e.g. regression coefficient) AND variation (e.g. standard deviation) or associated estimates of uncertainty (e.g. confidence intervals)
☒	☐	For null hypothesis testing, the test statistic (e.g. F, t, r) with confidence intervals, effect sizes, degrees of freedom and P value noted<br>*Give P values as exact values whenever suitable.*
☒	☐	For Bayesian analysis, information on the choice of priors and Markov chain Monte Carlo settings
☒	☐	For hierarchical and complex designs, identification of the appropriate level for tests and full reporting of outcomes
☐	☒	Estimates of effect sizes (e.g. Cohen's d, Pearson's r), indicating how they were calculated

Our web collection on statistics for biologists contains articles on many of the points above.

Software and code

Policy information about availability of computer code

Data collection	N.A.
Data analysis	N.A.

For manuscripts utilizing custom algorithms or software that are central to the research but not yet described in published literature, software must be made available to editors/reviewers. We strongly encourage code deposition in a community repository (e.g. GitHub). See the Nature Research guidelines for submitting code & software for further information.

Data

Policy information about availability of data

All manuscripts must include a data availability statement. This statement should provide the following information, where applicable:

- Accession codes, unique identifiers, or web links for publicly available datasets
- A list of figures that have associated raw data
- A description of any restrictions on data availability

Data supporting the findings of this manuscript are available from the corresponding author upon reasonable request. Atomic coordinates and structure factors have been deposited in the Protein Data bank under accession codes 6UZW (GluN1-GluN2A LBD - UBP791), 6UZX (GluN1-GluN2A-4m LBD - UBP791), 6UZ6 (GluN1-GluN2A-4m LBD - glycine/glutamate), 6UZR (GluN1-GluN2A LBD - HQA), and 6UZG (GluN1-GluN2A-4m LBD - HQA). Electrophysiology and cell assay data for Figures 2, 3, 6, 7 and Supplementary Figures S2, S4, S5 and Supplementary Table 2 are provided in the Source Data file.

Field-specific reporting

Please select the one below that is the best fit for your research. If you are not sure, read the appropriate sections before making your selection.

☒ Life sciences ☐ Behavioural & social sciences ☐ Ecological, evolutionary & environmental sciences

For a reference copy of the document with all sections, see nature.com/documents/nr-reporting-summary-flat.pdf

Life sciences study design

All studies must disclose on these points even when the disclosure is negative.

Sample size	X-ray diffraction data was collected until radiation damage of crystals weakened the diffraction data. Thus, sample size is determined by the experimental observation (not pre-determined). The typical number of redundancy of diffraction spots in this study is five or better.
Data exclusions	Oocyte recordings were excluded when there are artifacts associated with recording or oocyte health was compromised, as evidenced by drifting baseline or other problems.
Replication	Every electrophysiology experiment is conducted on at least five different oocytes.
Randomization	5-10% of random X-ray diffraction data in a given dataset was set aside and was used to test the molecular models generated by the rest of the data to calculate R(free) in the process of model refinement.
Blinding	Although not realistic to apply blinding to solve x-ray crytallographic structures, we would be able to distinguish one structure to another by checking the electron density of the ligand binding site. For example, we would be able to clearly tell differenced between the glutamate-bound structure and the UBP791-bound structures.

Reporting for specific materials, systems and methods

We require information from authors about some types of materials, experimental systems and methods used in many studies. Here, indicate whether each material, system or method listed is relevant to your study. If you are not sure if a list item applies to your research, read the appropriate section before selecting a response.

Materials & experimental systems

n/a	Involved in the study	
☒	☐	Antibodies
☒	☐	Eukaryotic cell lines
☒	☐	Palaeontology
☐	☒	Animals and other organisms
☒	☐	Human research participants
☒	☐	Clinical data

Methods

n/a	Involved in the study	
☒	☐	ChIP-seq
☒	☐	Flow cytometry
☒	☐	MRI-based neuroimaging

Animals and other organisms

Policy information about studies involving animals; ARRIVE guidelines recommended for reporting animal research

Laboratory animals	Xenopus laevis frogs were purchased from Nasco Scientific
Wild animals	N.A.
Field-collected samples	N.A.
Ethics oversight	Ethic oversight is conducted by American Association for Laboratory Animal Science

Note that full information on the approval of the study protocol must also be provided in the manuscript.
